# Elastic Properties and Energy Dissipation Related to the Disorder-Order Ferroelectric Transition in a Multiferroic Metal-Organic Framework [(CH_3_)_2_NH_2_][Fe(HCOO)_3_] with a Perovskite-Like Structure

**DOI:** 10.3390/ma14092403

**Published:** 2021-05-05

**Authors:** Zhiying Zhang, Xin Shen, Hongliang Yu, Xiaoming Wang, Lei Sun, Shumin Yue, Dongpeng Cheng, Hao Tang

**Affiliations:** 1 School of Materials Science and Engineering, Wuhan University of Technology, Wuhan 430070, China; shenxin1221@126.com (X.S.); yuhongliang2021@126.com (H.Y.); sunlei15171@163.com (L.S.); yueshumin1216@163.com (S.Y.); chengdongpeng93@163.com (D.C.); tanghao202103@163.com (H.T.); 2 Beijing National Laboratory for Condensed Matter Physics, Institute of Physics, Chinese Academy of Sciences, Beijing 100190, China; wangxiaomingteemo@163.com

**Keywords:** metal-organic framework (MOF), ferroelectric transition, X-ray diffraction (XRD), dynamic mechanical analysis (DMA), elastic property, energy dissipation

## Abstract

The elastic properties and the coupling of ferroelasticity with ferromagnetism and ferroelectricy are crucial for the development of multiferroic metal-organic frameworks (MOFs) with strong magnetoelectric coupling. Elastic properties and energy dissipation related to the disorder-order ferroelectric transition in [(CH_3_)_2_NH_2_][Fe(HCOO)_3_] were studied by differential scanning calorimetry (DSC), low temperature X-ray diffraction (XRD) and dynamic mechanical analysis (DMA). DSC result indicated the transition near 164 K. XRD showed the first-order structural transition from rhombohedral *R*$\overset{-}{3}$c to monoclinic *C*c at ~145 K, accompanied by the disorder-order transition of proton ordering in the N–H…O hydrogen bonds in [(CH_3_)_2_NH_2_]^+^ as well as the distortion of the framework. For single crystals, the storage modulus was ~1.1 GPa and the loss modulus was ~0.02 GPa at 298 K. DMA of single crystals showed quick drop of storage modulus and peaks of loss modulus and loss factor near the ferroelectric transition temperature ~164 K. DMA of pellets showed the minimum of the normalized storage modulus and the peaks of loss factor at ~164 K with weak frequency dependences. The normalized loss modulus reached the maximum near 145 K, with higher peak temperature at higher frequency. The elastic anomalies and energy dissipation near the ferroelectric transition temperature are caused by the coupling of the movements of dimethylammonium cations and twin walls.

## 1. Introduction

Metal-organic frameworks (MOFs) have attracted a lot of attention, and they have potential applications in gas storage, separation, catalysis, photoluminescence, sensors, magnetic and electric devices [1,2,3,4,5,6,7,8]. Multiferroic MOFs have drawn special interest due to tuneable properties and the coexistence of ferroelectricity/ferromagnetism/ferroelasticity [9,10,11,12,13,14,15]. In [NH_2_NH_3_][M(HCOO)_3_] (M = Mn, Fe, Co), electric phase transitions and structural phase transitions occur from non-polar *P*nma (No. 62) to polar *P*na2_1_ (No. 33) near 347 K (M = Fe, perovskite) and 350 K (M = Mn, perovskite), from polar *P*6_3_ (No. 173) to non-polar *P*2_1_2_1_2_1_ (No. 19) near 336 K (M = Fe, chiral) and 380 K (M = Co, chiral), or from polar *P*6_3_ (No. 173) to non-polar *P*2_1_ near 296 K (M = Mn, chiral), and magnetic phase transitions occur at 7.9–13.9 K (Mn: 7.9 K, Fe: 12.5 K, Co: 13.9 K) [9,10]. In [CH_3_NH_2_NH_2_][M(HCOO)_3_] (M = Mn, Fe), second-order structural phase transitions from nonpolar *R*$\bar{3}$c to polar *R*3c occur at 310 K, and first-order structural phase transitions occur at 180–225 K, and magnetic phase transitions occur at 9–21 K [11]. In [NH_4_][M(HCOO)_3_] (M = Mn, Fe, Co, Ni), electric phase transitions and structural phase transition from non-polar *P*6_3_22 to polar *P*63 occur at 191–254 K, and magnetic transitions occur at 8.4–29.5 K [12,13,14,15]. In [(CH_3_)_2_NH_2_][M(HCOO)_3_] (M = Mn, Fe, Co, Ni), electric phase transitions and structural phase transition from rhombohedral *R*$\bar{3}$c (No. 167) to monoclinic *C*c occur at 160–185 K, and magnetic transitions occur at 8.5–35.6 K with spin reorientation transitions at 13.1–14.3 K [16,17,18,19,20,21].

The elastic properties and the coupling of ferroelasticity with ferromagnetism and ferroelectricy are crucial for the development of multiferroic MOFs with strong magnetoelectric coupling [22,23,24,25,26,27]. There are a few reports about the elastic properties of [(CH_3_)_2_NH_2_][M(HCOO)_3_] (M = Mn, Co, Ni) [22,23,24,25,26,27]. The Young’s modulus of [(CH_3_)_2_NH_2_][M(HCOO)_3_] (M = Mn, Co, Ni) at room temperature was 19–25 GPa as determined by nanoindentation [22]. Resonant ultrasound spectroscopy (RUS) was used to investigate the elastic properties and the coupling of ferroelasticity with ferromagnetism and ferroelectricy in [(CH_3_)_2_NH_2_][M(HCOO)_3_] (M = Mn, Co, Ni) [23,24], and dynamic mechanical analysis (DMA) was used to study the elastic properties and the coupling of ferroelasticity with ferroelectricy in [(CH_3_)_2_NH_2_][Mn(HCOO)_3_] [25]. [(CH_3_)_2_NH_2_] [M(HCOO)_3_] (M = Fe) has received intensive attention due to its strong magnetoelectric coupling [28,29,30,31,32,33,34]. The elastic properties of [(CH_3_)_2_NH_2_][Fe(HCOO)_3_] have rarely been reported. Jain et al. pointed out due to twining, the low temperature XRD patterns of [(CH_3_)_2_NH_2_][M(HCOO)_3_] (M = Mn, Fe, Co, Ni) were difficult to be well refined [17]. Sanchez-Andujar et al. reported that the structure of [(CH_3_)_2_NH_2_][Mn(HCOO)_3_] at 100 K was monoclinic *C*c [35]. Maczka et al. reported that the structure of [(CH_3_)_2_NH_2_] [M(HCOO)_3_] (M = Fe) below ~160 K was monoclinic *C*c [32], but Ma et al. reported that the structure at 100 K was monoclinic *C*2/c [34].

The present work studied the temperature and frequency dependences of elastic properties of [(CH_3_)_2_NH_2_][Fe(HCOO)_3_] using DMA and investigated the elastic anomalies and energy dissipation associated with ferroelectric transition and structural transition with relaxation time 0.1–2 s. Our findings make contributions to the understanding of phase transition mechanisms.

## 2. Materials and Methods

### 2.1. Material Synthesis

Single-crystals of [(CH_3_)_2_NH_2_][Fe(HCOO)_3_] were prepared using a solvothermal method under the protection of N_2_ as reported in reference [17]. 5 mmol FeCl_2_·4H_2_O was dissolved in the mixture of 30 mL deionized water and 30 mL N,N-dimethylformamide, and the solution was placed in a Teflon-lined stainless-steel autoclave. The autoclave was kept at 413 K for 3 days and then slowly cooled to room temperature. Next, the supernatant was transferred into a tube and sealed in N_2_ filled environment. After 5 days, transparent crystals formed were collected by filtering the mother liquid and washing using ethanol. The crystals were kept in a desiccator.

### 2.2. Room Temperature Powder X-ray Diffraction (XRD)

Crystals of [(CH_3_)_2_NH_2_][Fe(HCOO)_3_] were ground to powder. A D8 Advance diffractometer (Bruker, Billerica, MA, USA) was used to collect the room temperature powder XRD. The diffraction angle 2θ was in the range of 10–60° and the step size was 0.02°. The Rietveld fit of XRD pattern was achieved through GSAS.

### 2.3. Differential Scanning Calorimetry (DSC)

A DSC 200F3 calorimeter (Netzsch, Selb, Germany) was used to perform the DSC measurements of [(CH_3_)_2_NH_2_][Fe(HCOO)_3_] crystals (around 16 mg) in a N_2_ filled environment from 140 K to 300 K at the heating rate of 5 K/min.

### 2.4. Low Temperature Powder XRD

A Smartlab powder XRD system (Rigaku, Tokyo, Japan) was used to collect the powder XRD patterns from 300 K to 10 K. 2θ was between 10° and 70°, and the step size was 0.02°. The temperature step of 20 K was set between 300 K and 180 K, step of 5 K between 180 K and 120 K, step of 20 K between 120 K and 20 K, and step of 10 K between 20 K and 10 K. The temperature accuracy was 0.1 K using a Lakeshore temperature sensor. Low temperature XRD patterns were refined using GSAS.

### 2.5. DMA

The temperature and frequency dependences of elastic properties and energy dissipation of [(CH_3_)_2_NH_2_][Fe(HCOO)_3_] single crystals were determined using DMA8000 instrument (PerkinElmer Instruments, Waltham, MA, USA) in the single cantilever mode from 150 K to 320 K at the rate of 2 K/min at frequencies of 1, 5 and 10 Hz.

Pellets of [(CH_3_)_2_NH_2_][Fe(HCOO)_3_] were obtained by pressing powder, as reported in reference [25]. The temperature and frequency dependences of elastic properties and energy dissipation of [(CH_3_)_2_NH_2_][Fe(HCOO)_3_] pellets were determined using a Diamond DMA (PerkinElmer Instruments, Waltham, MA, USA) in the compression mode from 130 K to 300 K at the rate of 2 K/min at frequencies of 0.5, 1, 2, 5 and 10 Hz. 

## 3. Results and Discussion

### 3.1. Room Temperature Powder XRD

**Figure 1:**
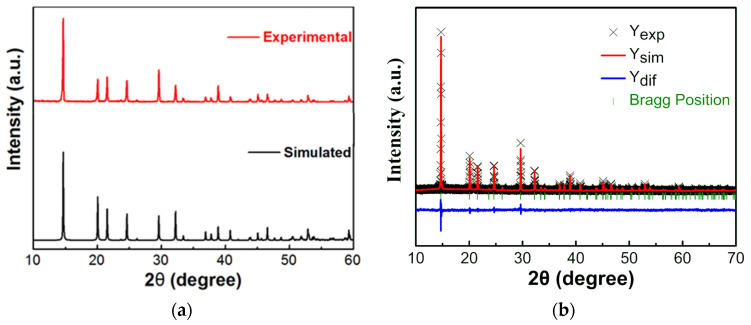
Powder XRD patterns of [(CH_3_)_2_NH_2_][Fe(HCOO)_3_] at room temperature, (**a**) the experimental and simulated XRD patterns, (**b**) Rietveld fit of XRD pattern. The simulated XRD pattern was for rhombohedral *R*$\overset{-}{3}$c structure (CCDC 780885) [17].

Figure 1 shows the experimental and simulation powder XRD patterns of [(CH_3_)_2_NH_2_][Fe(HCOO)_3_] at room temperature, as well as the Rietveld fit of XRD pattern. The experimental pattern is consistent with the simulation pattern of space group rhombohedral *R*$\bar{3}$c (CCDC 780885) [17]. The obtained lattice parameters α = β = 90°, γ = 120°, a = b = 8.2510(1) Å, c = 22.5611(7) Å, and the fitting parameters R_wp_ = 4.52%, R_p_ = 3.47% and χ^2^ = 1.648. 

**Figure 2:**
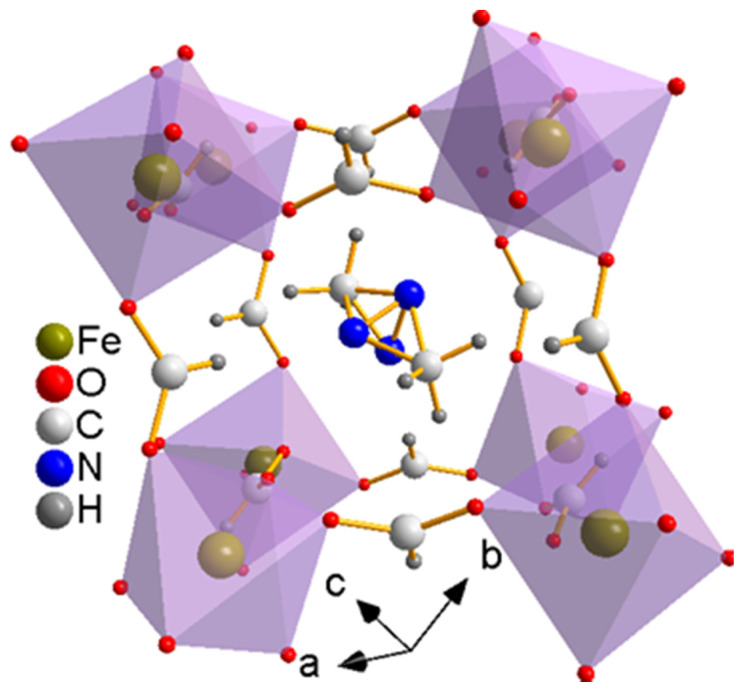
The rhombohedral *R*$\overset{-}{3}$c structure [17] of [(CH_3_)_2_NH_2_][Fe(HCOO)_3_] at room temperature.

They are in good agreement with the results reported by Jain et al. (α = β = 90°, γ = 120°, a = b = 8.241(2) Å, c = 22.545(6) Å [17]), the results reported by Zhou et al. (α = β = 90°, γ = 120°, a = b = 8.249 Å, c = 22.556 Å with R_wp_ = 4.849%, R_p_ = 3.462% and χ^2^ = 8.733 [33]), and the results reported by Ma et al. (α = β = 90°, γ = 120°, a = b = 8.2312(12) Å, c = 22.506(5) Å [34]). It was reported that the crystals usually grow along the [012] direction according to single crystal XRD analysis [28,29,30,31]. As shown in Figure 2, the structure of [(CH_3_)_2_NH_2_][Fe(HCOO)_3_] is ABX_3_ perovskite-like, in which A is [(CH_3_)_2_NH_2_]^+^, B is Fe^2+^, and X is HCOO^−^. The skeleton is formed by Fe^2+^ linked with HCOO^−^, and [(CH_3_)_2_NH_2_]^+^ is located in the central cavity. Hydrogen bonds are formed between the hydrogen atoms of [(CH_3_)_2_NH_2_]^+^ and the oxygen atoms of HCOO^−^.

### 3.2. DSC

**Figure 3:**
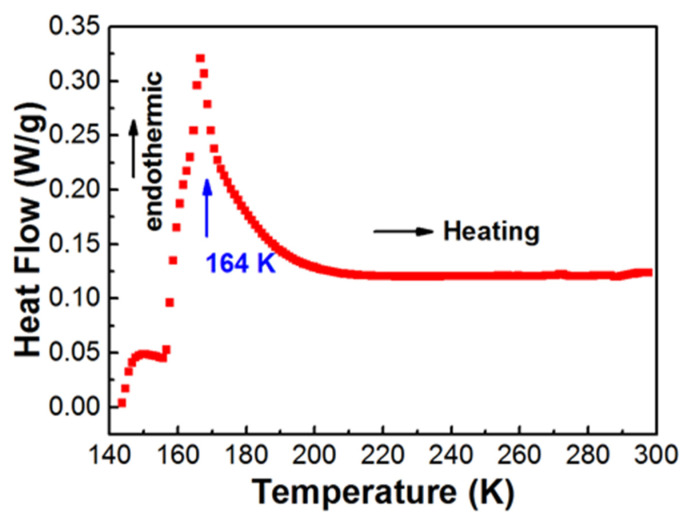
DSC curve of [(CH_3_)_2_NH_2_][Fe(HCOO)_3_] at the heating rate of 5 K/min.

As shown in Figure 3, DSC curve of [(CH_3_)_2_NH_2_][Fe(HCOO)_3_] from 140 K to 300 K indicated an endothermic peak at 164 K. The average enthalpy ∆H was 1202 J mol^−1^, and the average entropy ΔS was 7.2 J mol^−1^ K^−1^. The ratio of the configuration numbers in the disordered and ordered systems, N, was 2.35. N would be 3 for a simple 3-fold order-disorder model. Therefore, the transition in [(CH_3_)_2_NH_2_][Fe(HCOO)_3_] near 164 K was more complicated than a simple 3-fold order-disorder model for [(CH_3_)_2_NH_2_]^+^. For [(CH_3_)_2_NH_2_][Fe(HCOO)_3_], the temperature dependences of dielectric and pyroelectric properties showed a sudden jump near 164 K with a hysteresis in the transition temperature during heating and cooling processes [17,28,34], indicating a first order ferroelectric transition from paraelectric to antiferroelectric caused by the disorder-order transition of the hydrogen bonding. The polar hydrogen bonds were antiparallel in the antiferroelectric state [34]. Maczka et al. reported anomalies in the temperature dependences of Infra-Red and Raman spectra near 160 K [32], due to the first-order structural transition from rhombohedral *R*$\bar{3}$c at high temperature to monoclinic *C*c at low temperature, accompanied by the disorder-order transition of proton ordering in the N–H⋯O hydrogen bonds in [(CH_3_)_2_NH_2_]^+^ as well as the distortion of the metal-formate framework. Below the transition temperature, the ordered phase showed proton ordering in the N–H⋯O hydrogen bonds in [(CH_3_)_2_NH_2_]^+^ [32]. 

### 3.3. Low Temperature Powder XRD

**Figure 4:**
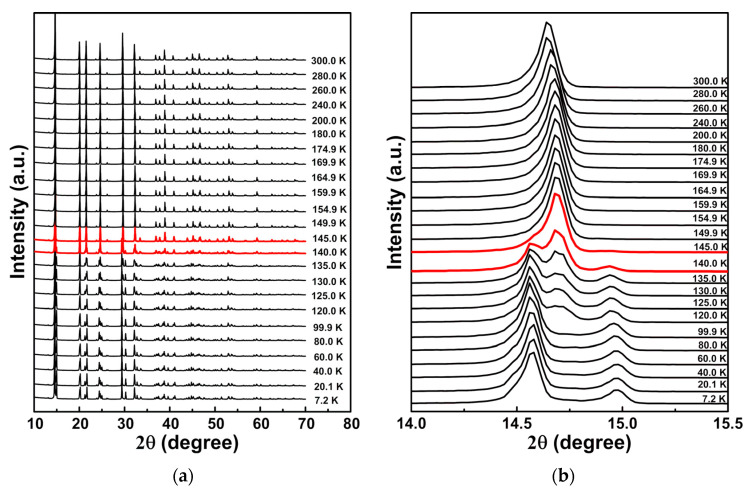

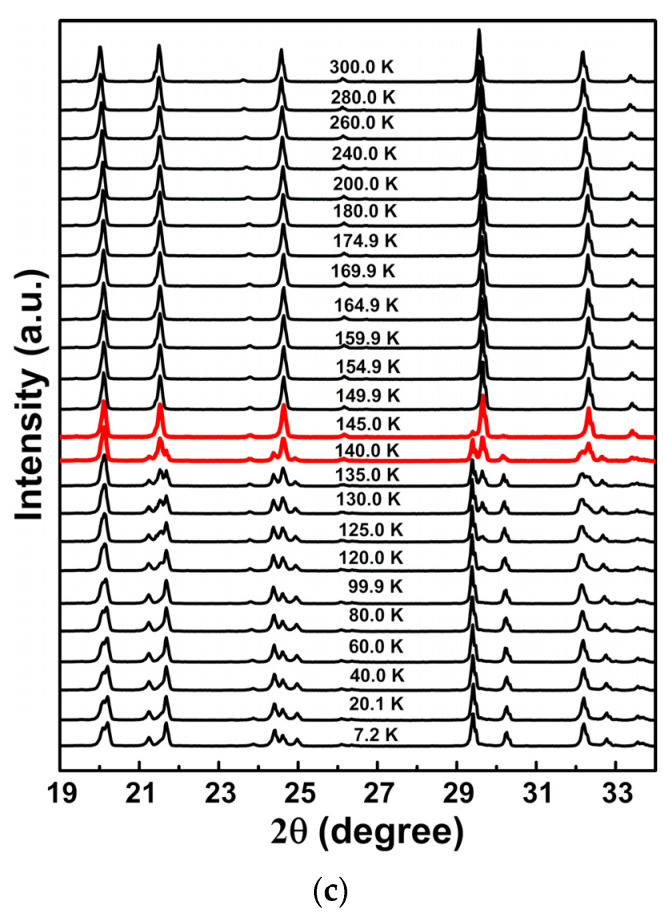
Powder XRD patterns of [(CH_3_)_2_NH_2_][Fe(HCOO)_3_] between 10 K and 300 K, with diffraction angle 2θ in the range of (**a**) 10–70°, (**b**) 14–15.5°, (**c**) 19–34°.

The powder XRD patterns of [(CH_3_)_2_NH_2_][Fe(HCOO)_3_] in the range of 10–300 K are shown in Figure 4. Structural transition occurred near 145 K. Compared with XRD patterns between 145 K and 300 K, XRD patterns between 10 K and 140 K show extra peaks with diffraction angle 2θ near 14.9°, 21.2°, 24.4°, 30.2°, 32.7°, 39.5° and 48.7°. The structure transition is from rhombohedral *R*$\bar{3}$c to monoclinic *C*c, which is associated with the cooperative ordering of the dimethylammonium cation, the tilting of the octahedral [FeO_6_], and the distortion of the flexible [Fe(HCOO_3_)]^−^ framework [17,32]. 

**Figure 5:**
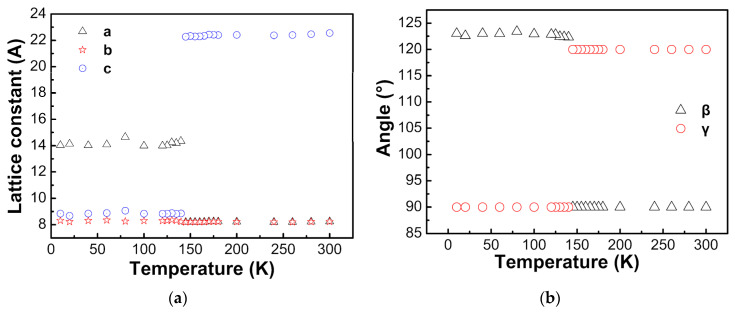
Temperature dependences of lattice parameters, (**a**) lattice constant, (**b**) angles.

The differences in the transition temperatures determined by DSC and low temperature XRD may be due to the different heating rates employed. The temperature dependences of lattice parameters obtained by Rietveld refinement of low temperature XRD patterns are shown in Figure 5. 

Above 145 K, Rietveld refinements of XRD patterns are consistent with rhombohedral *R*$\bar{3}$c with fitting parameters R_wp_ ≤ 11.19% and R_p_ ≤ 7.76%. Below 145 K, Rietveld refinements of XRD patterns are in agreement with monoclinic *C*c with fitting parameters R_wp_ ≤ 24.22% and R_p_ ≤ 15.97%. Jain et al. reported that due to twinning, XRD patterns of [(CH_3_)_2_NH_2_][M(HCOO)_3_] (M = Mn, Fe, Co, Ni) below the ferroelectric transition temperatures could not be well refined, but the low temperature structure was monoclinic [17]. For [(CH_3_)_2_NH_2_][Mn(HCOO)_3_] at 110 K, α = γ = 90°, β = 120.88(1)°, a = 14.451(8) Å, b = 8.376(3) Å, c = 8.952(4) Å, and for [(CH_3_)_2_NH_2_][Ni(HCOO)_3_] at 10 K, α = γ = 90°, β = 120.879(7)°, a = 14.451(8) Å, b = 8.376(3) Å, c = 8.952(4) Å [17]. Sanchez-Andujar et al. tried two possible space groups monoclinic *C*2/c (centrosymmetric) and *C*c (non-centrosymmetric) for the structure of [(CH_3_)_2_NH_2_][Mn(HCOO)_3_] at 100 K, and found that *C*c structure was better for the refinement, with β = 120.694(2)°, a = 14.345(2) Å, b = 8.323(1) Å and c = 8.879(1) Å [35]. [(CH_3_)_2_NH_2_]^+^ is different in the rhombohedral and monoclinic structures. C–O distances of all formate ions are the same in the rhombohedral structure, and they are slightly different in the monoclinic structures [35]. Ma et al. reported that for [(CH_3_)_2_NH_2_][Fe(HCOO)_3_], the structure at 120 K is monoclinic *C*2/c, α = γ = 90°, β = 122.81°, a = 14.100(3) Å, b = 8.3781(17) Å, c = 8.8983(18) Å [34]. 

### 3.4. DMA

The storage modulus E′ is the real part of the complex modulus of the viscoelastic material, and it is related to the elastic energy storage. The loss modulus E″ is the imaginary part of the complex modulus, and it is related to the internal energy dissipation. The loss factor tanδ is the ratio of the loss modulus to the storage modulus.

**Figure 6:**
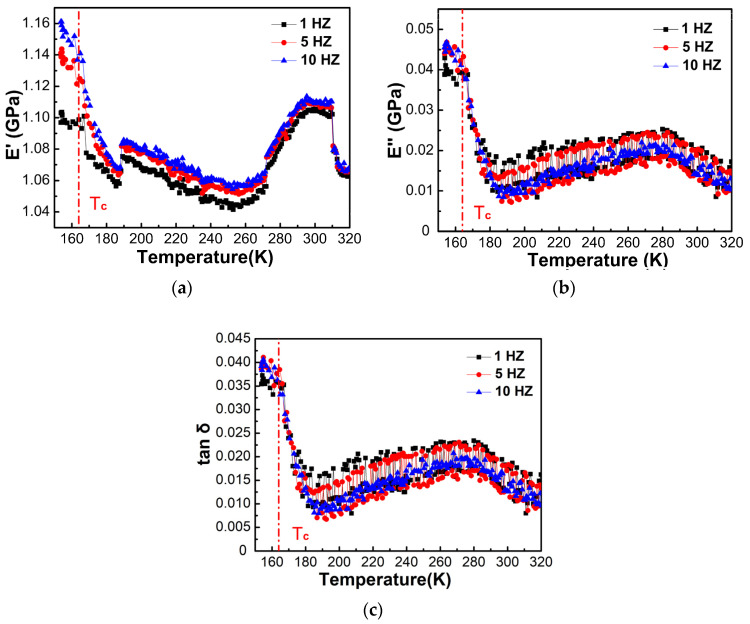
Temperature dependences of (**a**) storage modulus, E′, (**b**) loss modulus, E″, and (**c**) loss factor tanδ of [(CH_3_)_2_NH_2_][Fe(HCOO)_3_] single crystals at frequencies of 1–10 Hz determined by DMA during heating at the rate of 2 K/min. The vertical dash-dotted line indicates the ferroelectric transition temperature T_c_ ~ 164 K.

Figure 6 shows the changes of storage modulus E′, loss modulus E″ and loss factor tan δ of [(CH_3_)_2_NH_2_][Fe(HCOO)_3_] single crystals with temperature from 150 K to 320 K at frequencies of 1, 5, and 10 Hz. Near the ferroelectric transition temperature ~164 K, the storage modulus dropped quickly, and the loss modulus and loss factor reached the maximum. For [(CH_3_)_2_NH_2_][Fe(HCOO)_3_] single crystals, the storage modulus was ~1.1 GPa, the loss modulus was ~0.02 GPa, and the loss factor was ~0.015 near 298 K.

**Figure 7:**
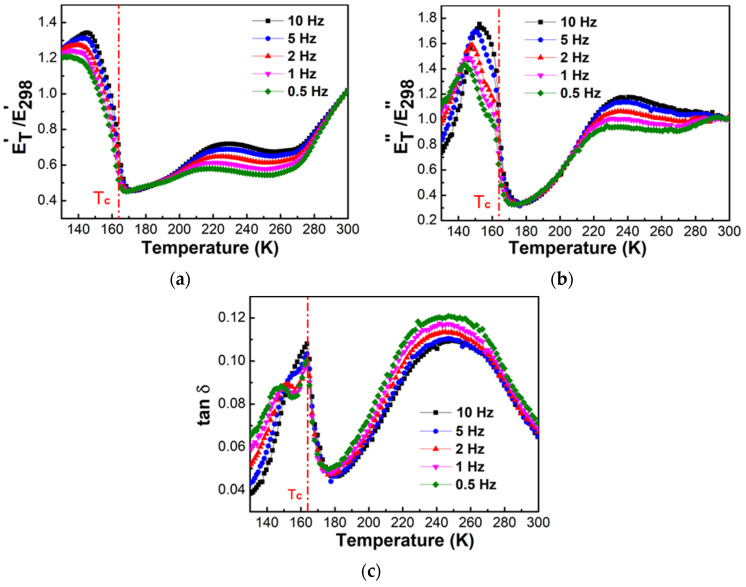
Temperature dependences of (**a**) the normalized storage modulus, E′_T_/E′_298_, (**b**) the normalized loss modulus, E″_T_/E″_298_, and (**c**) loss factor tan δ of [(CH_3_)_2_NH_2_][Fe(HCOO)_3_] pellets at frequencies of 0.5–10 Hz determined by DMA during heating at the rate of 2 K/min. The vertical dash-dotted line indicates the ferroelectric transition temperature T_c_ ~ 164 K.

For [(CH_3_)_2_NH_2_][Fe(HCOO)_3_] pellets, the storage modulus was ~55 MPa, the loss modulus was ~3.5 MPa, and the loss factor was ~0.065 near 298 K. Figure 7 shows the changes of the normalized storage modulus, i.e., the ratio of storage modulus at temperature T to that at 298 K, E′_T_/E′_298_, the normalized loss modulus, i.e., the ratio of loss modulus at T to that at 298 K, E″_T_/E″_298_, and loss factor tanδ of [(CH_3_)_2_NH_2_][Fe(HCOO)_3_] pellets with temperature from 130 K to 300 K at frequencies of 0.5, 1, 2, 5, and 10 Hz. The normalized storage modulus gradually dropped with the increase of temperature, from ~1.25 at 130 K to ~0.45 at ~164 K, and then gradually increased with temperature. The minimum in the normalized storage modulus occurred near the ferroelectric transition temperature ~164 K, and the softening reached ~64%. With the increase of temperature, the normalized loss modulus gradually increased and then decreased. The peak temperature for the normalized loss modulus was near 145 K, and the peak temperature increased with the increase of the frequency, from 144.5 K at 0.5 Hz to 152.4 K at 10 Hz. With the increase of the temperature, loss factor gradually increased and then decreased. The peak temperature for the loss factor was near the ferroelectric transition temperature ~164 K with weak frequency dependences, which is the feature of first-order phase transition. The peak height of the normalized loss factor and loss factor increased with the increase of the frequency. 

The anomalies near 280 K and 310 K in Figure 6 and the anomalies around 220–270 K in Figure 7 have no physical meaning, and they may be caused by the instability of the DMA measurements after the samples were under low frequency and high stress and strain conditions for some time.

**Figure 8:**
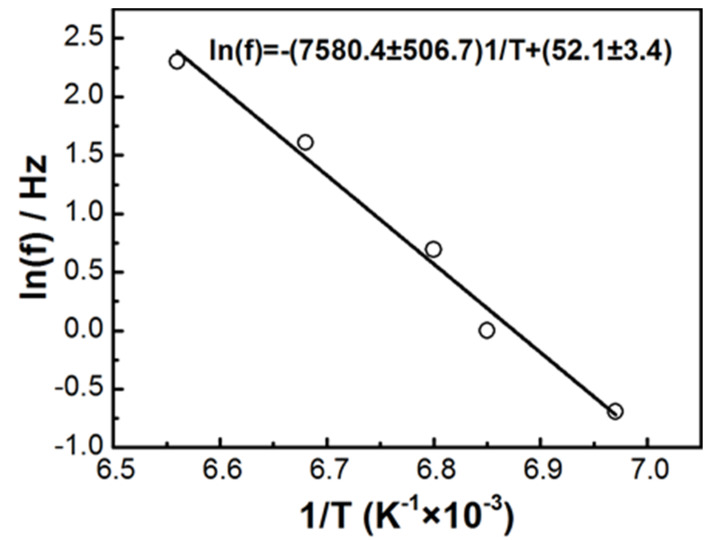
ln(f) vs. 1/T for the peaks of temperature dependences of E″_T_/E″_298_ near 145 K for [(CH_3_)_2_NH_2_][Fe(HCOO)_3_] pellets fitting by Arrhenius equation f = f_0_exp(−E_a_/RT).

Figure 8 shows the fitting of ln(f) vs. 1/T for the peaks of the temperature dependences of the normalized loss modulus near 145 K as shown in Figure 7b, using Arrhenius equation f = f_0_exp[−E_a_/(RT)], where R is the gas constant, T is the peak temperature for the normalized loss modulus near 145 K, and f is the frequency. The activation energy E_a_ was ~63 kJ/mol.

**Figure 9:**
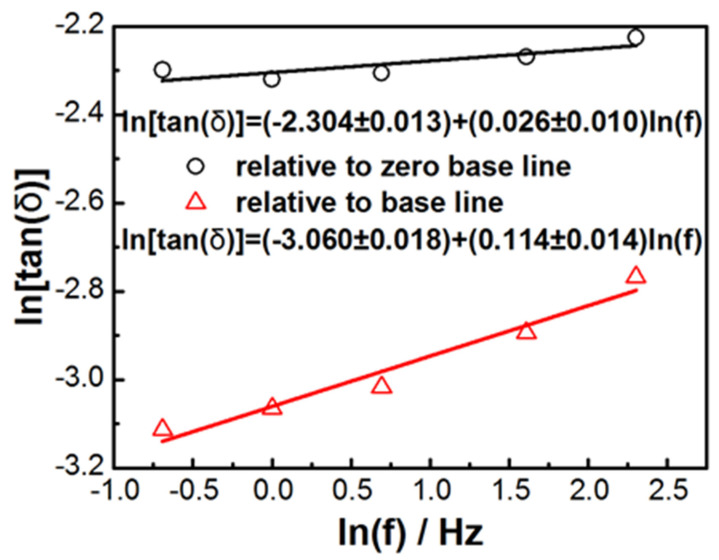
Double logarithmic plot ln(tan δ) vs. ln(f) for the peak height of tan δ near 164 K for [(CH_3_)_2_NH_2_][Fe(HCOO)_3_] pellets fitting by power law tan δ = Af^n^.

Figure 9 shows the fitting of the double logarithmic plot ln(tan δ) vs. ln(f) for the peak height of tan δ near 164 K as shown in Figure 7c, using power law tan δ = Af^n^, where A is the constance, and f is the frequency. 

The peak height of tan δ near 164 K was obtained using two methods. The first was relative to zero base line, i.e., no base line correction was used. The second was relative to the baseline, which was tangential to data points near 130 K and 180 K. n was determined to be between 0.026 and 0.114. The reported n value for [(CH_3_)_2_NH_2_][Mn(HCOO)_3_] was between −0.382 and −0.078 [25]. The elastic anomalies and energy dissipation detected by DMA near the ferroelectric transition ~164 K with relaxation time 0.1–2 s are due to the coupling of the movement of dimethylammonium cations and the mobility of twin walls. The relaxation processes in [(CH_3_)_2_NH_2_] [Fe(HCOO)_3_] pellets were similar to those reported for [(CH_3_)_2_NH_2_][Mn(HCOO)_3_] pellets investigated by DMA [25], with the minimum of storage modulus and the maximum of loss modulus and loss factor near the ferroelectric transition temperature ~190 K. For both E″ and tan δ of [(CH_3_)_2_NH_2_][Mn(HCOO)_3_], the peak temperature was independent of frequency, and the peak height decreased with the increase of the frequency. The relaxation processes in [(CH_3_)_2_NH_2_][Fe(HCOO)_3_] pellets were also similar to the reported dielectric spectroscopy analysis of [(CH_3_)_2_NH_2_][Fe(HCOO)_3_] [28], with the peaks of the dielectric constant and the dielectric loss near 164 K, and the increase in the peak temperature at higher frequency.

## 4. Conclusions

DSC analysis of [(CH_3_)_2_NH_2_][Fe(HCOO)_3_] from 140 K to 300 K showed an anomaly near 164 K, presumed to be due to the first order antiferroelectric to paraelectric transition accompanied by the first order structural transition from monoclinic to rhombohedral, related to the disorder-order transition of proton ordering in the N–H…O hydrogen bonds in [(CH_3_)_2_NH_2_]^+^. The ratio of the configuration numbers in the disordered and ordered systems, N, was 2.35, indicating that the transition in [(CH_3_)_2_NH_2_][Fe(HCOO)_3_] near 164 K was more complicated than a simple 3-fold order-disorder model for [(CH_3_)_2_NH_2_]^+^.

Low temperature powder XRD analysis of [(CH_3_)_2_NH_2_][Fe(HCOO)_3_] showed structural phase transitions near 145 K from monoclinic *C*c to rhombohedral *R*$\bar{3}$c related to the cooperative ordering of the dimethylammonium cation, the tilting of the octahedral [FeO_6_], and the distortion of the flexible [Fe(HCOO_3_)]^−^ framework. Due to twinning, the low temperature XRD patterns were not well refined. For [(CH_3_)_2_NH_2_][Fe(HCOO)_3_] single crystals, the storage modulus was ~1.1 GPa, and the loss modulus was ~0.02 GPa near 298 K from DMA measurements. They are lower than Young’s modulus of [(CH_3_)_2_NH_2_][M(HCOO)_3_] (M = Mn, Co, Ni) at room temperature, 19–25 GPa, determined by nanoindentation [22] due to the different loading methods between DMA and nanoindentation. DMA studies of [(CH_3_)_2_NH_2_][Fe(HCOO)_3_] single crystals and pellets showed elastic anomalies and large energy dissipation near the ferroelectric transition temperature ~164 K. Near the transition temperature, the normalized storage modulus reached the minimum, and the normalized loss modulus and the loss factor reached the maximum. The softening in normalized storage modulus reaches ~64%. When the frequency increases from 0.5 Hz to 10 Hz, the peak temperature for the normalized loss modulus increases from 144.5 K to 152.4 K, and the activation energy is ~63 kJ/mol. The peak temperature for the loss factor showed weak frequency dependences, which is a feature of first-order phase transitions. The elastic anomalies and energy dissipation are caused by the coupling of the movements of dimethylammonium cations and twin walls. 

The relaxation processes in [(CH_3_)_2_NH_2_][M(HCOO)_3_] (M = Fe, Mn) pellets through DMA were similar under low frequency and high stress and strain conditions, with relaxation time of 0.1–2 s, with strong coupling of ferroelasticity and ferroelectricity. Through RUS analysis, the relaxation processes in [(CH_3_)_2_NH_2_][M(HCOO)_3_] (M = Mn, Co, Ni) showed similar features under high frequency and low stress and strain conditions, with relaxation time of 10^−6^ s [23,24], and strong coupling of ferroelasticity, ferroelectricity and ferromagnetism were reported for [(CH_3_)_2_NH_2_][M(HCOO)_3_] (M = Ni) [24]. It is expected that RUS studies of the relaxation processes in [(CH_3_)_2_NH_2_][M(HCOO)_3_] (M = Fe) would also show strong coupling of ferroelasticity, ferroelectricity and ferromagnetism. Tailoring the strain, the metal ions and the hydrogen bonding may lead to the design of different functionality. The investigation of temperature dependences of elastic properties and elastic anomalies and energy loss related to ferroelectric transitions and magnetic transitions in MOFs are beneficial for the development of MOFs with good mechanical properties and strong coupling among ferroelasticity, ferroelectricity and ferromagnetism and their applications in sensors, data storage, magnetic and electric devices.

## Data Availability

The data presented in this study are available on request from the corresponding author. The data are not publicly available due to privacy.
